# Reflections: Surgical Education-the Times they are a-Changin’: Lessons Learned from the 3rd MAYMET-ESO Joint Meeting

**DOI:** 10.1007/s13187-015-0831-2

**Published:** 2015-04-24

**Authors:** Radoslaw Tarkowski, John T. Vetto

**Affiliations:** Department of Oncology, Division of Surgical Oncology, Wroclaw Medical University, pl. Hirszfelda 12, 53-413 Wroclaw, Poland; Division of Surgical Oncology, Oregon Health & Science University, 3181 S.W. Sam Jackson Park Rd, Portland, 97239 OR USA

## Abstract

Technical skills are not sufficient for successful surgical care. Non-technical skills such as team work, decision-making in cancer treatment, communication with the patient, ethical challenges, situation awareness, and communication in the operating room are mandatory for favorable outcomes. Although formally taught in other high-demand disciplines, such skills were traditionally rarely discussed in surgical oncology. The 3rd MAYMET-ESO Joint Meeting “Professionalism for Breast Surgeons” held in Istanbul, Turkey, 5 October 2013 was dedicated to the development of non-technical skills in the everyday activity of breast surgeons. We briefly discuss information from this very interesting and inspiring educational event and how it relates to more recent changes in surgical oncology education.

Come gather ‘round people

Wherever you roam

And admit that the waters

Around you have grown

And accept it that soon

You’ll be drenched to the bone

If your time to you

Is worth saving’

Then you better start swimming’

Or you’ll sink like a stone

For the times they are a-changin’.

Bob Dylan

One of us (RT) recently attended the 3rd MAYMET-ESO Joint Meeting entitled “Professionalism for Breast Surgeons” in Istanbul, Turkey in October 2013. He expected the “usual” technical surgical meeting, talks regarding assignment of patients to different oncoplastic interventions, problems of adverse events following surgery, and tips and tricks in the operating room (OR). Some surgeons believe that breast cancer operations represent a culmination of the treatment process performed on a surgical ward. Indeed, excellent surgery is crucial for favorable outcomes, especially in the treatment of patients with cancer, where suboptimal operations decrease survival rates.

In fact, participants at the meeting could find topics and displays only distantly related to surgical technique, clinical knowledge, hand dexterity, new equipment, and other technical topics that traditionally form the core of surgical curriculum. Instead, lectures given by noted breast surgeons encompassed *non-technical skills* such as communication with patients and colleagues, multidisciplinary team meetings, ethical challenges, situation awareness, decision-making, problem solving, disclosure of medical errors, leadership, and coping with stress (Table 1).

**Table 1:** The 3rd MAYMET-ESO Joint Meeting: “Professionalism for Breast Surgeons.” Summary of topics

Professional behavior in the preoperative period
Teamwork in diagnostic breast care
Non-technical skills for surgeons before surgery
Communications with patients and family: delivering bad news
Ethical challenges in treatment discussions and consent procedures
Surgeons and the OR
Situation awareness in the OR
Communication and teamwork in the OR
Non-technical skills for surgeons after surgery
Professional attitudes in the immediate postoperative period
Conveying effective oral and written information: reporting skills
How should the surgeon disclose medical error to the patient?
Leadership
Leadership in breast care
Understanding and managing stress in clinical settings

Non-technical skills, what Klampfer has called “observable, non-technical behaviors that contribute to superior or substandard performance” [1], are now known to have the same magnitude of impact on a surgical outcome as surgical technique, but, in contrast, until recently have been rather underestimated and all too missing in training programs. Data show that technical skills alone are not sufficient to ensure patient safety [2], that non-technical skills also have an impact on the technical performance of the surgeon [3], and that adverse events during surgery are often caused by a low quality of the non-technical aspects of practice. Although proper training in such fields exists in high demand professions other than surgery, particularly aviation [4], surgical oncologists’ education has traditionally concentrated on technique and experience—the latter mainly in terms of technical aspects of activity in ORs and clinics—while omitting non-technical skills [5]. One of us (JV) remembers a time not that long ago when communication and other non-technical skills were not only not taught in surgical training programs but were often viewed as unnecessary.

But the times they are a-changin’. A survey by Baldwin et al. showed an interesting breakdown of the skills identified by 68 Scottish surgeons as mandatory for successful outcomes: 29 (41 %) of the 70 skills named concerned communication, teamwork, and the application of knowledge, while 31 % (22/70) were clinical and only 27 % (19/70) were technical [6]. In a study by Gawande, communication breakdown (particularly during “hand-offs”) among personnel was identified as the cause of 43 % of errors in surgery, while excessive workload causing fatigue affected 33 % of errors. The authors concluded that more than half of the surgical errors could have been avoided with the proper use of non-technical skills [7]. Similarly, Rogers et al. demonstrated that communication breakdown was the cause of 25 % of adverse events resulting in lawsuits in surgery. Eleven percent were identified as problems during hand-offs, 9 % as a failure to establish clear lines of responsibility, and 11 % were attributed to poor communication between different members of the surgical team (particularly surgeons and nurses). Again, errors were often associated with fatigue and work overload, but the authors pointed to the use electronic medical records as a tool to reduce the rate of improper communication during hand-offs [8].

According to Lingard et al., preoperative checklists (such as the downloadable WHO form [http://www.who.int/patientsafety/safesurgery/ss_checklist/en/]) and team briefings can also reduce communication failures by about 34 %. Such tools identify gaps in knowledge and can lead to effective treatment changes [9]. Implementation of the above-mentioned tools in the daily practice of teams working in ORs is especially important, since about 50 to 75 % of adverse medical effects occur there [10]. When problems and errors do occur, it is critical that surgical team members discuss them in morbidity and mortality (and other structured) conferences without blaming or penalizing individuals. Instead, errors should be treated as a chance to improve quality, emphasizing the complex and interdependent relationships between different aspects of the surgical team critical for successful surgical outcomes [9, 10].

A non-technical skill gaining particular attention in ORs is situation awareness (SA), defined by Yule and Paterson-Brown as a three stage process: gathering information, interpreting information, and projecting and anticipating the future state of the patient based on the first two steps [11]. Surgeons, in particular, use SA to identify and understand information coming from several sources at once during a given surgical procedure (e.g. medical documentation, team members, display monitors, etc.) and then go one step beyond to analyze their possible consequences. In Rogers’ study of surgical lawsuits, 75 % of faults occurred during the intra-operative phase of treatment, and many were associated with barriers to SA (distracting noises, unnecessary chatter, loud music, talk on the radio, etc.) during the operation [8].

While many have pointed to parallels between aviation and surgery, these authors state that copying solutions from aviation and simply changing the word “*pilot*” into “*surgeon*” is not sufficient. Based on surgical education programs at the University of Aberdeen, Scotland which “observe, rate and provide feedback” on which of the aforementioned skills are desirable in the OR, Yule and colleagues have proposed the non-technical skills for surgeons (NOTSS) behavior rating system. NOTSS groups non-technical skills into four categories: SA, decision-making, communication and teamwork, and leadership (a “Task Management” category was later dropped after further analysis, and its elements were incorporated into the other categories) [12, 13].

“Bad news” is defined by Buckman as “any information which adversely and seriously affects an individual’s view of his or her future” [14]. At the time that one of us (JV) published a paper in this Journal on teaching how to break bad news [15], communication with cancer patients was thought by many to be an intrinsic skill that Oncologists did not need to be formally taught. Indeed, only a few members of the audience attending the “Breaking Bad News” session at the 3rd MAYMET-ESO Joint Meeting reported that they had previously taken part in any specialized training in breaking bad news, despite the fact that this is a key cancer education skill. Sadly, this was similar to the situation at the 1998 ASCO meeting 15 years before, where only 5 % of 400 oncologists surveyed reported receiving any training in this kind of communication, while 74 % said they did not have any specific approach, and 90 % reported emotional problems arising during the interview as a major barrier [16].

The long road cancer educators must still tread regarding teaching colleagues how to break bad news, and other cancer-related communication skills is no surprise; in the 1960s, 90 % of surgeons did not even discuss the diagnosis of cancer with their patients, choosing instead to “avoid stress” to the informed and the informers [17]. This approach changed by the late 1970s, when 90 % of physicians began to inform their patients about the diagnosis of cancer [18]. However, even then, cancer physicians were still woefully untrained at bad news and end of life discussions. With the number of interviews with a patient and their family in a 40-year career of a medical or surgical oncologist now estimated at 200,000 [8], the need for special training in communicating difficult and bad news is greater than ever. A study performed by Ptacek et al. revealed that the proportion of doctors who feel stressed after breaking bad news to their patients as 42 %, with bad effects on the bearer lasting up to 3 days afterwards [16]. A randomized study by Fallowfield et al. demonstrated that even experienced oncologists have difficulties in these areas that are not solved by their clinical experience, but that proper training of needed skills improves communication [19]. This suggests that experience and self-learning are not sufficient and must be augmented by specialized training.

And the times continue to change for the better. A formal emphasis on communication skills is at the center of the Accreditation Council for Graduate Medical Education (ACGME) Core Competencies, now an integral part of all U.S. surgical training programs [20]. Current surgical training includes formal teaching in patient communication skills, such as the S-P-I-K-E-S system (**s**etting up the interview, assessing the patient’s **p**erception, obtaining the patient’s **i**nvitation, providing **k**nowledge and information, addressing patients emotions with **e**mphatic responses, and **s**ummarizing the information and plan) [21] for delivering bad news. While S-P-I-K-E-S was stressed at the 3rd MAYMET-ESO Joint Meeting, it was particularly interesting to hear about unique issues regarding its application from participating experienced surgeons in countries with different patterns of professional interactions between patient and doctor (i.e., Sweden, Italy, UK, Netherlands, UK, and Turkey). Meeting discussion also stressed how S-P-I-K-E-S can help distinguish the differing patient information needs and desire to be touched or not touched during the S-P-I-K-E-S encounter, both of which are examples of using cultural sensitivity in communication [6].

Communication is central to teamwork, and the 3rd MAYMET-ESO Joint Meeting included a session on communication between cancer team members, both in the OR (where stress can be a particular barrier [22]) and during multidisciplinary team meetings and tumor boards, which are crucial in maintaining the highest quality of treatment (and have been a research focus of this Journal for years [23]). Another session of the meeting concerned leadership, an area where good communication skills can make the difference between being a manager and a leader in a medical setting.

Coping with stress, another non-technical skill, is particularly important in surgery, which produces everyday exposure to emotional stress, fatigue, and work overload. Stress can even impair hand dexterity and cognitive processes and their integration. In a study by Hull et al., higher levels of stress caused increased numbers of errors [2]. Further, stress impairs teamwork by changing the perspective from team level to self level, resulting in poor cohesion between team members and poorer outcomes [24]. Hull measured intra-operative stress levels (using a dedicated psychological test) in different members of OR teams and found them to be higher among surgeons than anesthetists. Stress was the highest in assisting (resident and fellow) surgeons, possibly resulting from a lack of self-confidence and the presence of senior colleagues [2, 25]. These authors concluded that evidence-based training of non-technical skills will lead to the creation of a generation of surgeons who are competent in the skills necessary for effective treatment of patients [2]. Indeed, several non-technical skill modules are already included in surgical education programs in the USA, such as the American College of Surgeons and the Association of Program Directors in Surgery (ACS-APDS) Surgical Skills Curriculum [26] and some of the previously mentioned ACGME competencies [20].

The times in Surgical Oncology education are indeed (finally) a-changin’, and this was symbolized by the fact that the MAYMET-ESO conference took place in Istanbul, which is in the Bosphorus land bridge joining Europe and Asia. Surgery is an art that encompasses much more than technical skills; teaching and developing non-technical skills will make the surgical oncologist a far more effective provider. Like the Bosphorus, the event was like a bridge joining these two pillars of surgical skill.

## Abbreviations

ACGME: Accreditation Council for Graduate Medical Education
ACS-APDS: American College of Surgeons and the Association of Program Directors in Surgery
ESO: European School of Oncology
MAYMET: Marmara Anadolu Yakasi Meme Hastaliklari Sürekli Egitim Toplantilari
MDT: Multidisciplinary team
NOTSS: Non-technical skills for surgeons
OR: Operation room
SA: Situation awareness
